# Lewis Y Promotes Growth and Adhesion of Ovarian Carcinoma-Derived RMG-I Cells by Upregulating Growth Factors

**DOI:** 10.3390/ijms11103748

**Published:** 2010-09-29

**Authors:** Feifei Li, Bei Lin, Yingying Hao, Yan Li, Juanjuan Liu, Jianping Cong, Liancheng Zhu, Qing Liu, Shulan Zhang

**Affiliations:** 1 Department of Obstetrics and Gynecology, Shengjing Hospital of China Medical University, Shenyang, 36 Sanhao Street, Heping, Shenyang, 110004, China; E-Mails: faye.f.lee@gmail.com (F.L.); nymph77cn@yahoo.com.cn (Y.H.); liujuanjuan_2000@yahoo.cn (J.L.); congjianping2009@126.com (J.C.); feifei_1120@yeah.net (L.Z.); liuqing_111@126.com (Q.L.); zhangsl2010@yeah.net (S.Z.); 2 Department of Obstetrics and Gynecology, Provincial Hospital Affiliated to Shandong University, Jinan, China; 3 Department of Obstetrics and Gynecology, Maternity and Children Healthcare Center of Shenyang, Shenyang, China; E-Mail: snowy2005@live.cn (Y.L.)

**Keywords:** Lewis y, ovarian cancer, proliferation, tumorigenecity, adhesion, inhibition

## Abstract

Lewis y (LeY) antigen is a difucosylated oligosaccharide carried by glycoconjugates on the cell surface. Overexpression of LeY is frequently observed in epithelial-derived cancers and has been correlated to the pathological staging and prognosis. However, the effects of LeY on ovarian cancer are not yet clear. Previously, we transfected the ovarian cancer cell line RMG-I with the α1,2-fucosyltransferase gene to obtain stable transfectants, RMG-I-H, that highly express LeY. In the present study, we examined the proliferation, tumorigenesis, adhesion and invasion of the cell lines with treatment of LeY monoclonal antibody (mAb). Additionally, we examined the expression of TGF-β1, VEGF and b-FGF in xenograft tumors. The results showed that the proliferation and adhesion *in vitro* were significantly inhibited by treatment of RMG-I-H cells with LeY mAb. When subcutaneously inoculated in nude mice, RMG-I-H cells produced large tumors, while mock-transfected cells RMG-I-C and the parental cells RMG-I produced small tumors. Moreover, the tumor formation by RMG-I-H cells was inhibited by preincubating the cells with LeY mAb. Notably, the expression of TGF-β1, VEGF and b-FGF all increased in RMG-I-H cells. In conclusion, LeY plays an important role in promoting cell proliferation, tumorigenecity and adhesion, and these effects may be related to increased levels of growth factors. The LeY antibody shows potential application in the treatment of LeY-positive tumors.

## 1. Introduction

Ovarian cancer is the most lethal gynecologic malignancy. Ovarian cancer at its early stages is difficult to diagnose until it spreads and advances to later stages. Invasion and metastasis are the leading cause for poor prognosis and death. Studies have found that glycoconjugates on the cell membrane are closely related to cellular biological characteristics such as cell adhesion, invasion and metastasis [1–3]. Lewis y (LeY) is a difucosylated oligosaccharide with the chemical structure [Fucα1→2Galβ1→4(Fucα1→3)GlcNAcβ1→R], belonging to A, B, H, Lewis blood group antigens family. Under normal physiological conditions, LeY is mainly expressed during embryonic development. In adults, its expression is confined to the epithelium and granulocytes [4]. However, LeY antigen is expressed in most epithelial-derived cancer cells, among which 70–90% shows an increased expression of LeY antigen, including colon, stomach, ovary, breast and lung cancer [5–9]. In breast and lung cancer patients, the expression of LeY antigen correlates positively with poor prognosis [8,9].

α1,2-fucosyltransferase (α1,2-FT) is the key enzyme in LeY synthesis. Previously, we transfected the ovarian cancer cell line RMG-I with human α1,2-FT gene (FUT1) to obtain stable transfectants, RMG-I-H, that highly express LeY [10,11]. Our studies showed that, compared with cells without transfection, RMG-I-H cells have enhanced malignant behavior, a shorter cell cycle, and increased resistance to 5-fluorouracil. [10,12] suggesting that LeY is involved in the changes in biological behavior of the RMG-I-H cells.

LeY is a potential therapeutic target for LeY-positive cancers. Anti-LeY mAb have been shown to have excellent specificity and potential therapeutic value in the treatment of prostate [13], breast [14,15] and small cell lung cancer [16]. However, there is no such evidence showing their effects on ovarian cancer. In the present study, we compared the changes in biological behavior of RMG-I-H cells after LeY mAb treatment in order to demonstrate the function of LeY antigen and to provide a theoretical basis for the biological treatment of ovarian cancer. In addition, we examined the expression of TGF-β1, VEGF and b-FGF in nude mouse xenograft tumors. We found that the expression of all three cytokines was upregulated in RMG-I-H cells. This, on the one hand, provides a possible mechanism for the increased proliferation and adhesion in RMG-I-H cells; on the other hand, provides a basis for future studies on the role of LeY in signal transduction.

## 2. Results

### 2.1. Expression of LeY Increases on the Cell Surface after Transfection of the α1, 2-FT Gene

**Figure 1:**
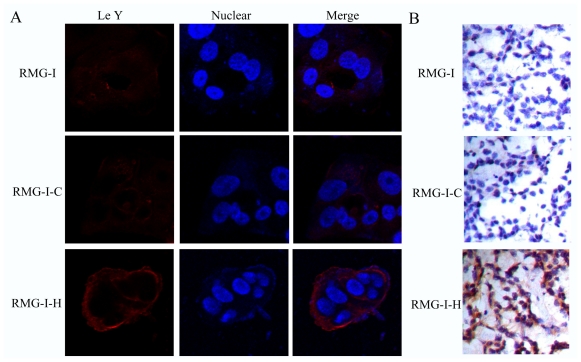
Determination of expression of LeY. (**A**) Cells were fixed and stained for LeY using LeY mAb labeled with Rhodamine (TRITC)-conjugated AffiniPure Goat Anti-Mouse IgG (red). Blue: Nuclei stained with 4,6-diamidino-2-phenylindole (DAPI); (**B**) Immunohistochemistry for LeY in xenograft tumors.

Previous results showed that transfection of the α1, 2-FT gene into RMG-I cells resulted in a 20–30 fold increase in cellular α1, 2-FT activity [10]. We here verify the expression of LeY on cells. Laser confocal microscopy revealed that the expression of LeY on the surface of RMG-I-H cells was obviously higher than in RMG-I and RMG-I-C cells (Figure 1A). Immunohistochemical assays were performed to examine the expression of LeY in nude mouse xenograft tumors formed from subcutaneous injection with RMG-I, RMG-I-C and RMG-I-H cells. Similarly, the results showed that RMG-I-H cells had a higher expression of LeY than the two control groups (*P* < 0.05) (Figure 1B), indicating that the α1, 2-FT gene transfection increased the expression of LeY.

### 2.2. LeY Enhances Proliferation of RMG-I Cells

**Figure 2:**
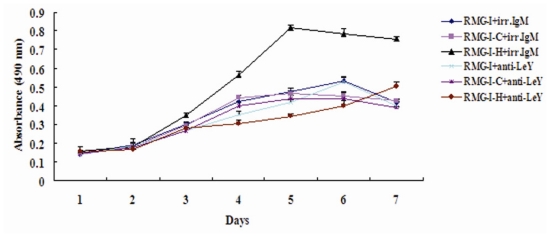
Cell proliferation was determined by the MTT assay. Cells treated with either anti-LeY mAb or control IgM antibody were incubated with MTT to perform cell growth analysis. Data are means ± SE from three individual experiments.

Since proliferation effectively reflects the malignancy of tumors, studying proliferation is an important way to understand the biological behavior of tumors. Using the MTT method, we detected absorbance values (490 nm) of cells at different time points after treatment with LeY mAb or the control IgM antibody. The results showed that the proliferation in LeY mAb-treated groups all decreased compared with that in the control IgM antibody-treated groups, among which the LeY mAb-treated RMG-I-H cells had the most obvious change (*P* < 0.05) (Figure 2). The inhibition rates of proliferation from day 1 through day 7 were 3.13%, 5.59%, 19.71%, 45.73%, 57.80%, 48.92% and 33.38%, respectively. The highest inhibition rate was on day 5, which was close to 60%. These results indicate that elevated expression of LeY led to an increase in the number of RMG-I cells when they were cultured *in vitro*.

### 2.3. LeY Enhances Tumorigenicity of RMG-I Cells

**Figure 3:**
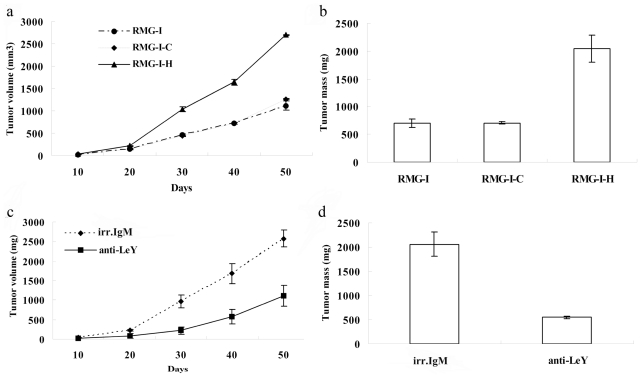
LeY increases tumor growth in nude mice. 15 nude mice divided into 3 groups were injected with RMG-I, RMG-I-C or RMG-I-H cells [10^7^ cells/0.3 mL]. During the inhibition assay, LeY mAb and irrelevant IgM antibody treated RMG-I-H cells [10^7^ cells/0.3 mL] were subcutaneously injected into nude mice. Tumor volume (**A**, **B**) and tumor mass (**C**, **D**) were assessed as mentioned in the Materials and Methods section.

This experiment was performed to determine the tumorigenic ability of the cell lines in nude mice. The results showed that the latent period of tumor growth was 8.8 ± 1.3 d and 8.6 ± 0.5 d in RMG-I cells and RMG-I-C cells, respectively, with no significant difference (*P* > 0.05), whereas the latent period of tumor growth was 5.2 ± 0.8 d in RMG-I-H cells, significantly less than that in the control groups (*P* < 0.01). The xenograft tumor volume was significantly larger in the RMG-I-H group compared to the control groups (*P* < 0.01) (Figure 3A). Similarly, tumor weight in the RMG-I-H group (2038.8 ± 241.9 mg) was also significantly higher than in the control RMG-I (696.8 ± 73.3 mg) and RMG-I-C (707.2 ± 21.8 mg) groups (*P* < 0.01) (Figure 3B). However, we found no difference between the RMG-I cells and RMG-I-C cells in growth potential *in vivo*. To further determine if tumor formation by RMG-I-H is dependent on LeY antigen, cells were inoculated after preincubation with LeY mAb. The results shown in Figures 3 (Figures 3C and 3D) illustrate that tumor formation was significantly inhibited by LeY mAb. These results demonstrate that LeY antigen enhances the tumorigenicity of RMG-I cells *in vivo*.

### 2.4. LeY Enhances Adhesion of RMG-I Cells

**Figure 4:**
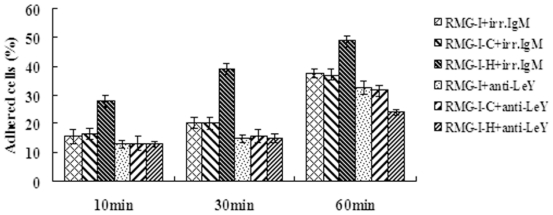
Changes in cell adhesion after anti-LeY mAb/irrelevant IgM antibody treatment. Data are means ± SE from three individual experiments.

This experiment was performed to compare the adhesion of cells with or without LeY mAb treatment. Inverted microscopy revealed that cells in LeY mAb treated groups failed to spread or spread more slowly, with shorter and smaller cell bases according to morphological analysis. After the same culture time, the number of adhered cells in LeY mAb-treated RMG-I-H groups was decreased obviously compared with that in the control IgM antibody-treated groups (*P* < 0.05) (Figure 4), and the inhibition rates of adhesion were 53.17%, 61.50% and 50.93% at 10, 30 and 60 min, respectively. The results indicate that the LeY antigen is involved in the adhesion of ovarian cancer RMG-I cells.

### 2.5. LeY Does Not Affect Cell Invasion of RMG-I Cells

RMG-I, RMG-I-C and RMG-I-H cells treated with different concentrations of LeY mAb (1 μg/mL, 5 μg/mL, 10 μg/mL and 20 μg/mL) were subjected to invasion assays using a transwell cell culture system. Matrigel were coated on the upper face of the transwell. The results suggested that there was no detectable difference in invasion between LeY mAb treated groups and control antibody treated groups (*P* > 0.05).

### 2.6. LeY Upregulates the Expression of TGF-β1, VEGF and b-FGF

**Figure 5:**
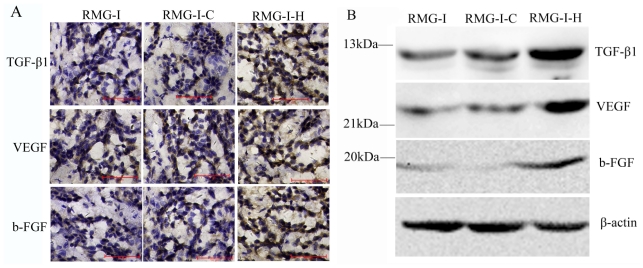
Determination of the expression of TGF-β1, VEGF and b-FGF. (**A**) Expression of TGF-β1, VEGF and b-FGF in nude mouse xenograft tumor tissues using immunohistochemical methods; (**B**) Expression of TGF-β1, VEGF and b-FGF in cells cultured *in vitro* using western blot analysis.

VEGF, b-FGF and TGF-β1 are growth factors with multiple biological functions that play important roles in enhancing tumor cell proliferation, angiogenesis and tumor development [17–19]. To gain insight into the mechanism for enhanced proliferation, tumorigenicity and adhesion by LeY antigen, we examined the expression of TGF-β1, VEGF and b-FGF in both nude mouse xenograft tumors and cells cultured *in vitro* by immunohistochemical staining and Western blot analysis, respectively. The results showed that the expression of TGF-β1, VEGF and b-FGF were all increased in RMG-I-H cells (*P* < 0.05) (Figure 5A and 5B), suggesting that the LeY is likely to enhance tumor cell proliferation and adhesion by promoting the secretion of these cell growth factors.

## 3. Discussion

Based on previous studies, we treated cells before and after transfection with LeY mAb in order to block the function of LeY antigen on the cell surface. *In vitro* proliferation and adhesion assays showed that the LeY mAb significantly inhibited the proliferation and adhesion of RMG-I-H cells. More importantly, we tested cell growth *in vivo* by inoculating subcutaneously the cells into nude mice. While the RMG-I cells and RMG-I-C cells with low level of LeY expression on the cell surface produced small tumors, LeY high-expressing cells, RMG-I-H, grew large tumors. LeY-dependent growth of RMG-I-H cells *in vivo* was supported by the following results: The size and weight of tumors derived from RMG-I-H cells were reduced significantly by preincubation of RMG-I-H cells with anti-LeY mAb. We preliminary found that the lactose type I chain family of the original RMG-I cells were Lc4Cer, Lewis a, and Lewis b, whereas, H-1 instead had the absolute domination in RMG-I-H cells. For the glycolipids of the lactose type II chain family, such as LeX, LeY, IV3NeuAc-nLc4Cer and NeuAc-LeX, their concentrations were over 0.01 μg per milliliter of dry cells; however, the glycolipids shown in RMG-I-H cells were LeX and LeY. 42.6% of LeX in the RMG-I-H was converted into LeY, which was in much higher percentage than the 3.2% of the original RMG-I cells. Although type I chain family H-1 had the absolute domination in the transfected RMG-I-H cells, its actual content was only a quarter of the LeY [10]. These results further proved that the changes in biological behavior of RMG-I-H cells had to do with the increase in LeY antigen. All these results indicate that the LeY antigen is related to increased cell proliferation, adhesion and tumorigenecity of RMG-I-H cells.

LeY causes significant enhancement of proliferation and growth possibly due to the following reasons. First, LeY structure was found being contained within the epidermal growth factor receptor (EGFR) on the surface of tumor cells and to participate in EGFR signaling [20–22]. Second, LeY antigen is likely an effective mediator to promote angiogenesis. VEGF and b-FGF are two powerful angiogenic cytokines. Zhu *et al*. [23] studied the role of LeY/H antigen in angiogenesis via *in vitro* experiments using a sugar analog (H-2g) of the LeY-6/H-5-2 (LeY/H) antigen. The results showed that H-2g activated NFκB through the JAK2 and the PI3K pathways, which induced the expression of VEGF and bFGF, thereby mediating angiogenesis. Previously, we used gene chip techniques to detect the differential expression of tumor-related genes after α1, 2-FT gene transfection [24]. The results showed 88 differentially expressed genes after transfection, among which VEGFB gene expression was up-regulated. In the present study, we demonstrated that the expression of VEGF and bFGF increased in ovarian cancer RMG-I-H cells, providing further evidence that there are important links between the LeY antigen and angiogenesis. Furthermore, our recent study showed that the TGF-β receptors also contain the structure of LeY. The amount of LeY in the structure of TGF-β receptors was significantly increased in RMG-I-H cells, and the TGF-β-dependent activation of the MAPK and the PI3K signaling pathways was enhanced (unpublished work [25]).

Zhang *et al.* [26] studied the association between surface glycan structure and biological behaviors of human hepatoma cell line 7721 using a-L-fucosidase to remove terminal fucosyl residues on the cell surface. Their results suggested that fucose is involved in adhesion. LeY is mainly distributed at the plasma membrane of cancer cells, and carried by different glycolipids and glycoproteins. Studies showed that changes in fucosyltransferase expression might affect LeY structure on cell surface receptors and therefore impact the expression and function of some adhesion-related glycoprotein receptors, such as CD44v6 [27], ICAM-2 [28] and integrins [29]. In the present study we demonstrate by *in vitro* adhesion assay that LeY mAb can efficiently inhibit the adhesive ability of RMG-I-H cells, further demonstrated that LeY is involved in adhesion of RMG-I cells. In addition, we used matrigel to establish an artificial basement membrane and determined their invasive potential by testing the number of tumor cells able to penetrate the membrane. The results showed that the effect of LeY on the invasion of ovarian cancer cells was not significant. However, the results may be related to the antibody used, so further experiments are required.

## 4. Materials and Methods

### 4.1. Reagents and Cell Lines

Mouse anti-human LeY mAb (clone A70-C/C8) [30] was purchased from Abcam (England). Rhodamine (TRITC)-conjugated AffiniPure goat anti-mouse IgG (H+L) was purchased from Zhongshan Biotechnology (China). Mouse anti-human IgM antibody, MTT reagents and 4,6-diamidino-2-phenylindole (DAPI) were purchased from Sigma (U.S.). The transwell (two vertical chambers separated by an 8 μm layer of polycarbonate membrane) was purchased from Corning (US). Matrigel was from BD Biosciences (US). Rabbit anti-human VEGF, rabbit anti-human b-FGF and rabbit anti-human TGF-β1 polyclonal antibodies were from Santa Cruz (US). The immunohistochemical SABC kit was purchased from Boster Biological Technology, Inc. (Wuhan, China).

The RMG-I cell line, which was originated from ovarian clear cell carcinoma, donated by Professor Iwamori Masao of Tokyo University of Japan. RMG-I-H is the cell line with a high expression of α1,2-FT and LeY antigen, and was established as previously reported [10,11]. RMG-I-C was the cell line transfected with the vector alone. The cells were cultured in DMEM (high-glucose) media (Invitrogen, Carlsbad, CA) containing 10% FBS (Clark, Australia) at 37 °C in a 5% CO_2_ incubator.

### 4.2. Laser Confocal Microscopy

The cells were seeded on a slide and fixed with 4% paraformaldehyde. The mouse anti-human LeY mAb was diluted at 1:100 as the working primary antibody; the goat anti-mouse TRITC fluorescence-conjugated secondary antibody was diluted at 1:200 as the working secondary antibody. After blocking with normal goat serum for 30 min, the cells were incubated with the working primary antibody at 37 °C for 1 h and then left at 4 °C overnight. After washing with PBS, the cells were incubated with the working secondary antibody at 37 °C for 100 min. DAPI was then used to stain the nuclei at room temperature for 5 min. The stained slide was observed under a laser confocal microscope (C1-SI; Nikon, Tokyo, Japan). Data were collected with a computer to generate digital images.

### 4.3. Cell Proliferation Assay

The cells were seeded in a 96-well plate at 2 × 10^4^ cells/well (three replicates), incubated at room temperature for 2 h, and then cultured in DMEM (with 10% FCS) containing LeY mAb (10 μg/mL) as previously described [31]. Mouse anti-human IgM antibody was added as control. Cells were cultured in 5% CO_2_ at 37 °C for 72 h, and then 20 μL of 5 mg/mL MTT was added. After 5 h of subsequent culture, 150 μL of DMSO was added to terminate the reaction, and the absorbance value was detected at 490 nm using a microplate reader (Elx808; Bio-Tek Instruments, Vermont, USA).

### 4.4. Subcutaneous Xenograft Tumor Model in Nude Mice

Ethical approval was obtained from China Medical University animal ethics committee before the start of the study. Fifteen 5–6 week old healthy female nude mice (Balb/c *nu/nu*, purchased from Liaoning Experimental Animal Center) were randomized into three groups and given a dorsal subcutaneous injection with RMG-I, RMG-I-C or RMG-I-H single cell suspensions (10^7^ cells/0.3 mL). In the inhibition experiments, 10^7^ cells were first preincubated with LeY mAb or IgM antibody (10 μg/mL) as previously described [31]. Cells were then washed with PBS and resuspended in 0.3 mL of serum-free DMEM media. These cells were incubated subcutaneously as described above. Animals were raised under SPF conditions and their general status was periodically monitored. The length (*a*), width (*b*) and height (*c*) of the tumors were measured by Vernier caliper, and the approximate tumor volume (*V*) was calculated by the formula *V* = π/6(*abc*). For small tumors, the height (depth) was roughly equal to the smaller value between *a* and *b*. The nude mice were all sacrificed 50 days later, and the tumors were isolated and weighed. The tumor tissues were then fixed in 10% formaldehyde, embedded in paraffin and cut into sequential slices (4 μm).

### 4.5. Cell Adhesion Assay

LeY mAb (10 μg/mL) and the control IgM antibody were added to single-cell suspensions, respectively. The cells were incubated at 37 °C for 30 min and then seeded into Petri dishes (35 mm) at 3 × 10^5^ cells/dish. Three dishes were randomly placed into each group after 10, 30, and 60 min of culture, and were then washed with PBS to remove non-adherent cells. Adherent cells were digested off and counted. The inhibition rate (*IR*) was calculated by the following formula:

IR=[1-(adherent cells in experimental groups/adherent cells in control groups)]×100%

### 4.6. Cell Invasion Assay

The bottom of the upper chamber was covered with a layer of matrigel to form a bottom barrier and dried overnight. 600 μL of 20% cell culture medium was added to the lower chamber, and 100 μL of cell suspension (1 × 10^6^/mL) was added to the upper chamber. After 2 h of incubation in 5% CO_2_ at 37 °C, LeY mAb was added to both the upper and the lower chambers (at a final concentration of 1, 5, 10 or 20 μg/mL). Cells that did not migrate through the membrane were removed, and the cells that migrated to the lower face of the membrane were fixed with methanol followed by staining with Giemsa. The numbers of cells on the lower face were counted using a high-power field under microscope. There were three replicates at each concentration from each group, and the experiment was repeated three times.

### 4.7. Immunohistochemical Staining

The tissue sections were processed by the general deparaffinization and hydration procedure, and were then soaked in freshly prepared 3% hydrogen peroxide for 10 min. After antigen retrieval, the tissue sections were incubated in normal goat serum for 30 min. Primary antibodies (1:100) were then added and incubated at 4 °C overnight. Biotin-conjugated secondary antibodies were added, followed by 20 min incubation at 37 °C. The SABC reagent was then added, followed by 30 min incubation at 37 °C. The sections were developed by DAB and monitored under the microscope. After counterstaining with hematoxylin, the slides were dehydrated, cleared, mounted, and observed under a microscope.

### 4.8. Western Blot Analysis

Cells were rinsed with PBS, and 1% of Triton X-100 lysis buffer (20 mM Tris-HCl, pH 7.4, 10 mM EGTA, 10 mM MgCl_2_, 1 mM benzamidine, 60 mM β-glycerophosphate, 1 mM Na_3_VO_4_, 20 mM NaF, 2 μg/mL aprotinin, 5 μg/mL leupeptin, 0.1 mM phenylmethylsulfonyl fluoride) was added. The suspension was centrifuged, and the supernatants were collected. Protein content was measured using the protein assay BCA kit (Beyotime biotechnology, China), and equal amounts of protein were loaded on SDS-PAGE gels. Subsequently, proteins were transferred to PVDF membranes (Millipore, Beaford, MA) and were probed with antibodies (1:1000). Immunoreactive bands were visualized by chemiluminescence (ECL; Pierce) using a secondary antibodies (1:8000).

### 4.9. Statistical Analysis

The SPSS 12.0 statistical analysis software was used, and the analysis of One-Way ANOVA was employed. *P* < 0.05 was regarded as with statistical significance.

## 5. Conclusions

Our study demonstrates that LeY antigen plays an important role in promoting the development of ovarian cancer, and that the LeY antigen may affect the behavior of ovarian cancer cells by upregulating the secretion of TGF-β1, VEGF and b-FGF.
